# Semi-Automated Graphical System for Calculating Pulmonary Vascular Impedances in a Clinical Setting

**DOI:** 10.1109/OJEMB.2021.3076726

**Published:** 2021-05-06

**Authors:** Timothy N. Bachman, Kang Kim, Marc A. Simon

**Affiliations:** Department of BioengineeringUniversity of Pittsburgh 6614 Pittsburgh PA 15261 USA; Department of Bioengineering and Department of MedicineUniversity of Pittsburgh 6614 Pittsburgh PA 15261 USA; Department of Bioengineering and the HeartVascular Institute of University of Pittsburgh and University of Pittsburgh Medical Center 6595 Pittsburgh PA 15261 USA

**Keywords:** Afterload, characteristic impedance, hemodynamics, pulmonary vascular impedance, total pulmonary resistance, vascular stiffness

## Abstract

*Goal:* Create a semi-automated, graphical, stand-alone application that uses clinically available asynchronous pressure and Doppler velocity captures to rapidly calculate, display, and interpret the pulmonary vascular impedance (PVZ) spectra. *Methods:* MATLAB-based software was written to analyze PVZ by creating a composite PVZ (cPVZ) spectra comprised of asynchronous screen captures of pulmonary arterial pressure and pulmonary arterial pulsed-wave Doppler velocity waveforms obtained during standard of care procedures. The pressure waveform, Doppler frequency envelopes, and ECG signals were re-digitized via automated border detection. cPVZ of averaged representative beats was calculated in the frequency domain via Fast Fourier Transform, and plotted vs harmonic z. *Results:* Successful generation of impedance spectra (PVZ(z)), where z is the harmonic, and additional parameters for characteristic impedance (Zc) and stiffness (Zs) were calculated as the mean of PVZ(2-4), and the sum of PVZ (1, 2), respectively. *Conclusions:* A graphically driven analysis of PVZ, calculated from standard of care right heart catheterization and echocardiography is possible. This system can help characterize both the steady and pulsatile components of right ventricular (RV) afterload in the clinical setting.

## I. Introduction

Overload of the right ventricle (RV) frequently leads to heart failure and death. Therefore, characterizing the full afterload encountered by the RV is critical. The pulmonary vascular impedance (PVZ) spectrum, graphed in the frequency domain, is the only way to fully characterize the combined steady and pulsatile afterload faced by the right ventricle (RV), providing novel insights for diagnosis and treatment [1], [2]. Analysis of PVZ to characterize the ventricular afterload has been performed for many decades in the research setting [3]–[7]. Unfortunately, current technical and cost limitations allow only the steady component (pulmonary vascular resistance (PVR) or total pulmonary resistance (TPR)) to be reported clinically. We have created a proof-of-concept, graphical, stand-alone application that uses semi-automated steps and clinically available asynchronous pressure and Doppler velocity captures to calculate, display, and compile the PVZ spectra and its resulting parameters for clinicians and researchers.

## II. Materials and Methods

**Figure 1.**
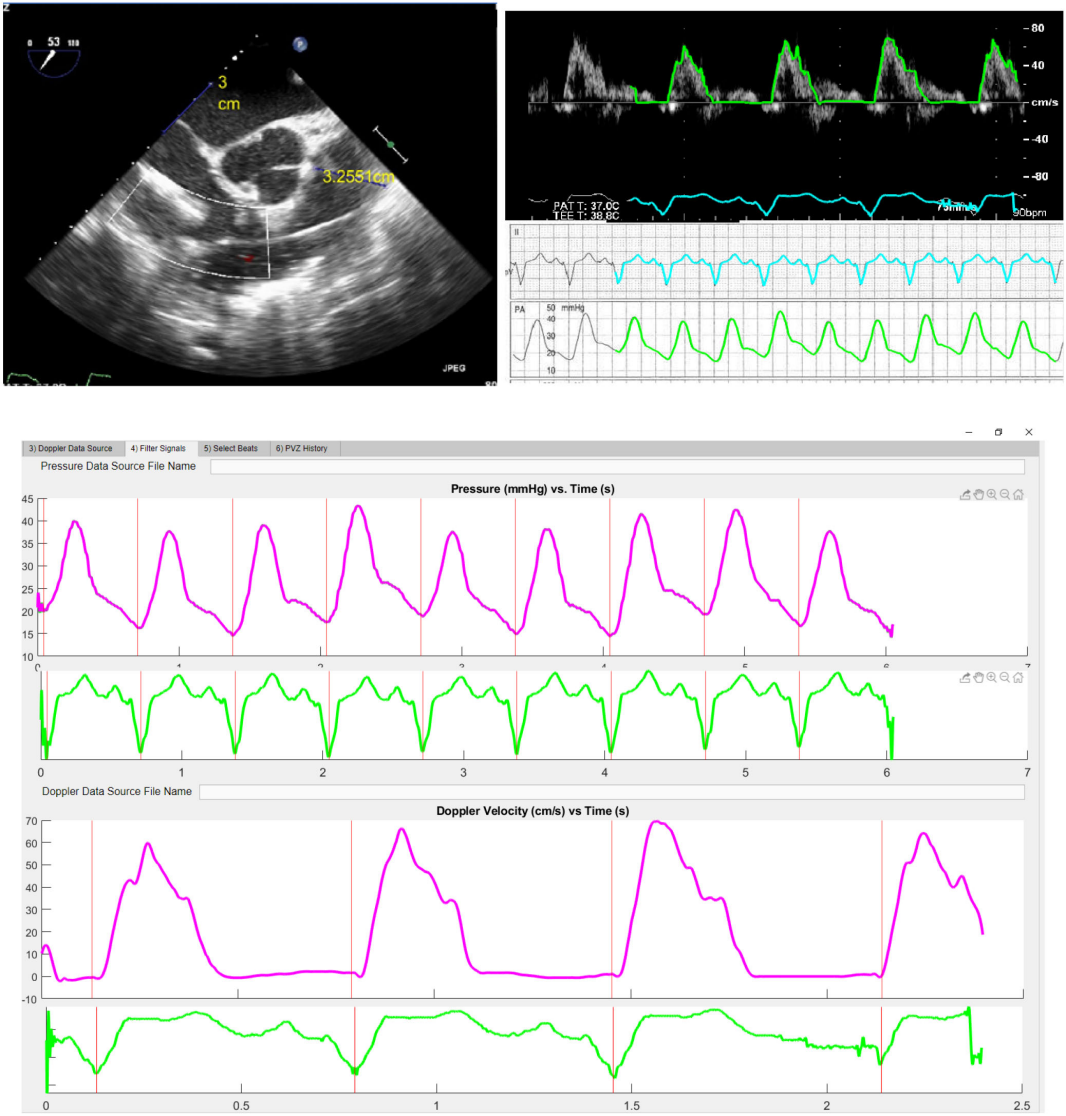
(Top left) Parasternal short axis view of the main pulmonary artery (PA). PA diameter is measured at the pulmonic valve. (Top Right) automated tracing of pulsed-wave Doppler velocity in the proximal PA and PA pressures along with ECG for timing. (Bottom) Individual beats for both pressure and Doppler velocity are identified using ECG signals from each capture.

Our MATLAB-based CompositePVZ© software (Fig. 1) analyzes a patient's PVZ and its changes during a study using screen captures obtained asynchronously during the following standard of care procedures: 1) Pulmonary artery (PA) diameter measurement and resulting cross-sectional area (CSA) measured at the pulmonic valve from a short axis view obtained via transesophageal echocardiography (TEE) or transthoracic echocardiography (TTE). 2) Recordings of PA pulsed-wave Doppler velocity in the proximal PA, V(t), where t equals time, from either TTE or TEE. 3) PA pressure signals (P(t)) obtained during standard right-heart catheterization.

**Figure 2.**
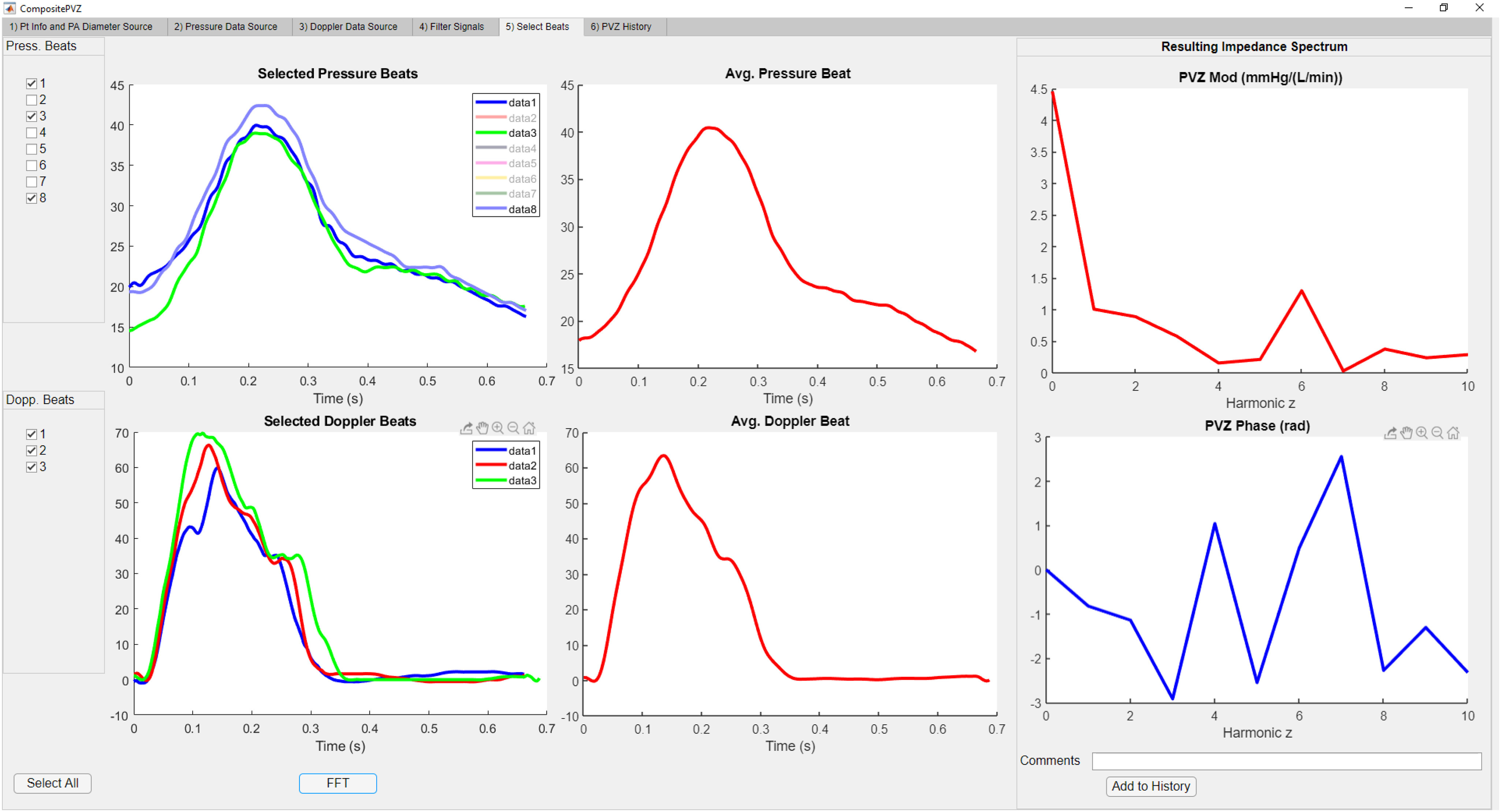
(Left) Individual beats selected to form average representative beat (center). Once completed for pressure and Doppler waveforms, the waveforms are decomposed via FFT. The ratio of pressure to flow in the frequency domain yields a PVZ spectra consisting of PVZ modulus and Phase (right).

As previously reported, our group has used semi-automated waypoint-driven tracing of waveforms to re-digitize retrospective images of waveforms [8]. Unfortunately, this can be time consuming. To remedy this problem, we have now developed an automated border detection function that identifies waveforms and frequency envelopes. Following re-digitization, the function resamples the waveforms at 1000 Hz. The product of V(t) and CSA is calculated to obtain flow (Q(t)). Individual beats separated by R-R interval of the ECG signal are selected to generate average representative beats (ARBs) for both P(t) and Q(t). The ARBs are then decomposed into harmonics (z) via Fast Fourier Transform (FFT), giving us P(z) and Q(z) in the frequency domain. Modulus and phase difference of PVZ, calculated as the ratio of P(z) to Q(z) regardless of actual frequency, are plotted versus harmonics z(n) (Fig. 2); where z(0) is the mean value of the signal, z(1) is the fundamental frequency (reported in Hz as the inverse of heart rate), and higher harmonics z(2) through z(N) are multiples of z(1). N is the maximum frequency from which PVZ can be determined by the fidelity of the catheter signals. (see components listed in Table 1). We have determined that conventional fluid-filled Swann-Ganz Catheters are reliable up to the 3^rd^ harmonic [9]. Huez et al were able to obtain reliable data at even higher harmonics with conventional catheterization [6].

**TABLE 1:** Components of PVZ and Resulting Parameters

Symbol	Parameter	Description
*z*	Harmonic	z= 0 is the steady component; z=1 is the fundamental frequency calculated as the inverse of heart rate. z=2…N are multiples of z=1
*PVZ(z)*	Pulmonary vascular impedance	Magnitude of the ratio of pressure flow in the frequency domain
Z0	Total pulmonary resistance	PVZ(0) Impedance at the zeroth harmonic
*Zc*	Characteristic Impedance	Mean impedance of PVZ(2) through PVZ(4)
*Zs*	Stiffness	Sum of PVZ(1) and PVZ(2)
		

## III. Results

**Figure 3.**
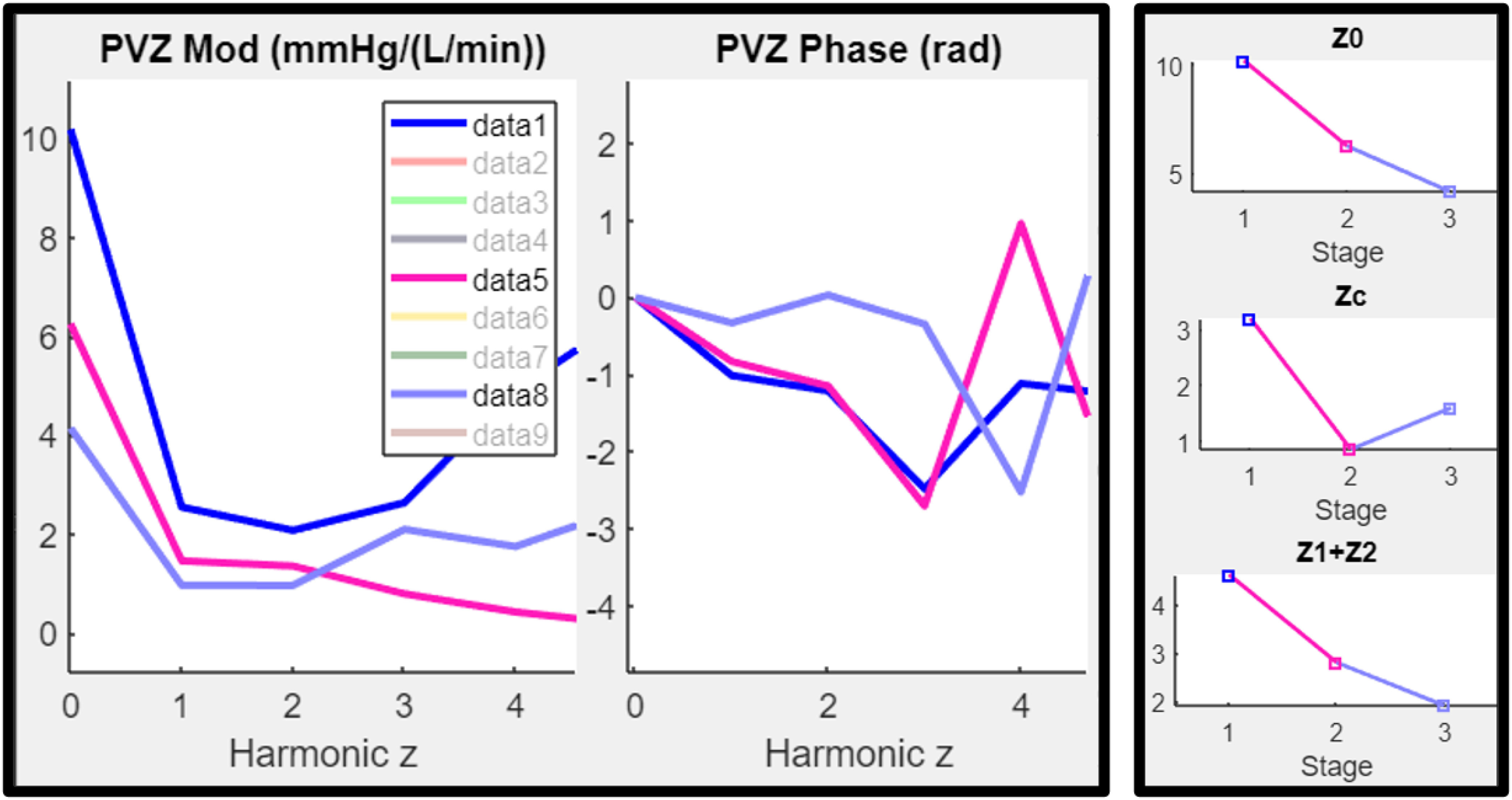
(Left) Overlay of pulmonary vascular impedance spectra for a single subject, taken at three different stages of a left ventricular assist device (LVAD) implant. (Stage 1) Pre-LVAD with patient conscious. (Stage 2) Patient in operating room anesthetized pre- implant. (Stage 3) Patient anesthetized post implant with LVAD unloading the LV. (Right) comparison of parameters derived from the three spectra. Video of analysis process for this figure can be viewed online (see supplementary material).

Fig. 3 shows the resulting overlay of PVZ at three timepoints of a study involving the same subject under at three stages of left ventricular assist device (LVAD) implantation. (Stage 1) Pre-LVAD with patient conscious. (Stage 2) Patient anesthetized pre- implant. (Stage 3) Patient anesthetized post implant with reduction in PVZ. Additional parameters that are calculated include: characteristic impedance (Zc) which is the average of PVZ(2-4) and stiffness (Zs) which is the sum of PVZ (1 and 2), see Table 1. The investigator can acquire multiple captures and choose which to include and exclude, similar to cardiac output calculations in the catheterization lab. Parameters from selected spectra are then plotted and can be exported as a table for further analysis. A supplemental flow chart of the analysis process and a video which provides a demonstration of the software is available online. (see supplementary material).

## IV. Discussion

A graphically driven analysis of PVZ, calculated from standard of care right heart catheterization and echocardiography is possible. Our graphical user interface is designed to be similar to the catheterization software already found in the catheterization lab. The similarities will enable clinicians to rapidly select captures of pressure, flow, and resulting spectra to assess the afterload faced by a patient's RV. However, due to the asynchronous manner in which the data is obtained, care should be taken to minimize time between TTE or TEE and right heart catheterization and to ensure that heart rates and rhythms are similar to avoid error. Future versions will also facilitate variable Zc harmonics as high-fidelity wires become more accessible. Multiple potential clinical applications at advanced heart failure and pulmonary hypertension centers exist including drug studies [10], exercise studies, and evaluation of surgical interventions.

## V. Conclusion

In conclusion, a rapid, graphically driven system for calculation of PVZ is feasible. A system such as this will allow full characterization of the RV afterload in the clinical setting, providing clinicians with a tool to characterize patients with pulmonary vascular disease and likelihood of RV failure.

## VI. Supplemental Materials

A flow diagram of the steps required for PVZ analysis using CompositePVZ© software can be found in the supplemental materials section online. Also, a video of the full analysis which resulted in Fig. 3 is available online.

A flow diagram of the steps required for PVZ analysis using CompositePVZ© software

A video of the full analysis which resulted in Fig. 3 is available online.
