# Supplementary figures and images for: Semi-Automated Graphical System for Calculating Pulmonary Vascular Impedances in a Clinical Setting
Source: IEEE Open J Eng Med Biol. 2021 May 6;2:198–200. doi: 10.1109/OJEMB.2021.3076726 (PMC8979623; doi:10.1109/OJEMB.2021.3076726)

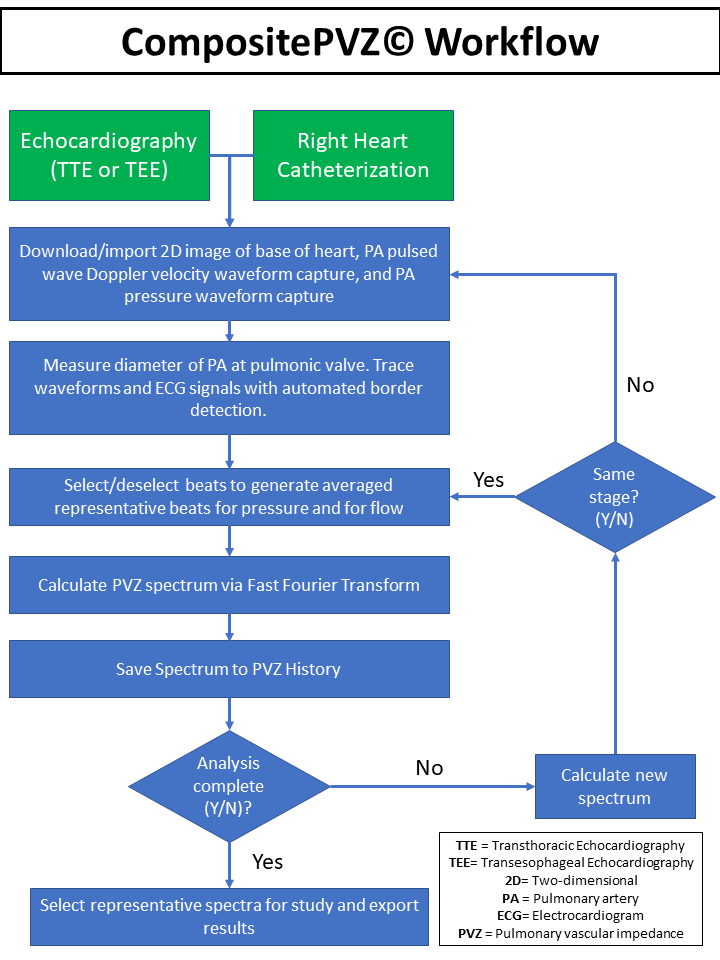

Supplement: A flow diagram of the steps required for PVZ analysis using CompositePVZ© software [file supp3-3076726.png]
